# The American Medical Association

**Published:** 1885-05

**Authors:** 


### EDITORIAL.

### THE AMERICAN MEDICAL ASSOCIATION.

The recent annual meeting of the American Medical Association, in New Orleans, presented a remarkable contrast to the meeting held in the Crescent city sixteen years ago. The number of physicians in attendance was large,—about eight hundred,—and thoroughly representative. Texas, alone, contributed a contingent of over one hundred. The reception given to the members of the Association by the physicians of New Orleans was cordial and hospitable, in the extreme, contrasting sharply with the entertainment in 1869,—when the "desolated homes and shattered fortunes demanded too much of the time of many Southern physicians to permit their attendance, while others had not sufficiently buried war memories to clasp hands in cordial greeting with their brothers of the North, so that many of each section failed to be present." The receptions of Dr. and Mrs. T. G. Richardson, Mr. and Mrs Cartwright Eustis, the members of the New Louisiana Jockey Club, on Wednesday evening, and of the medical profession, assisted by the ladies of New Orleans, on Thursday evening, were pleasant occasions, reminding us vividly of the words of a great physician: "The parts and signs of goodness are many. If a man be gracious and courteous to strangers, it shows he is a citizen of the world, and that his heart is no island cut off from other lands, but a continent that joins to them."

Dr. Henry F. Campbell, the President, in his address, alluded to "the father of the Association," and the editor of the *Journal of the American Medical Association*, in the following words:

"Too full of the meekness of wisdom to be elated by praise.

too worthy of praise to be defiled by flattery, and among all the living, standing foremost in the duration and constancy of his devotion, I could ask you to allow me, by virtue of the authority in which I am clothed, to bid him stand, as sovereigns bid their heroes kneel, and dub him with the ensign and the title, '*Noblest Roman of us all.*' "

Dr. Campbell's recommendation that a committee be appointed to consider the expediency of organizing, or of rehabilitating a section of forensic medicine, was favorably received, and a committee was selected.

*The Journal of the American Medical Association* will continue to be published in Chicago, under the editorship of Dr. N. S. Davis.

The work of the Sections, all things considered, was highly creditable.

The numerous papers, read before the Section on Obstetrics and Diseases of Women, were the result of the activity of the officers of the Section, Dr. R. S. Sutton, of Pennsylvania, and Dr. J. T. Jelks, of Arkansas.

*The Medical News*, one year ago, urged the importance of allowing each Section to select its own presiding officers, instead of relegating the work to the General Nominating Committee.

On Thursday, Dr. W. W. Potter, of Buffalo, N. Y., called up the amendment to the by-laws, offered last year by Dr. Foster Pratt, of Michigan, authorizing this change. After considerable discussion, the amendment was laid over for consideration next year. The utterly inadequate representation of the profession in certain Sections at the last meeting contains a pointed moral

We earnestly hope that all who are sincerely concerned for the welfare of the Association will urge the adoption of Dr. Foster Pratt's amendment, at the next meeting, in St. Louis.

